# The Detection of Renal Calculi by Aid of Radiography

**Published:** 1899-06-03

**Authors:** 


### THE DETECTION OF RENAL CALCULI BY AID OF RADIOGRAPHY.

In an address delivered before the Oxford Medical Society on May 12th, Dr. Mansell Moullin insisted on the superiority of the screen over the plate in the detection of abnormal conditions in deeply-buried parts. Nothing, he said, was easier than to obtain a representation of a thin structure, such as the hand. The difficulty was to obtain a picture of structures lying at considerable depths, whose absorptive index might not be very different from that of the tissues which surrounded them, and in such cases he thought it was much better to make a thorough investigation with the screen before attempting to take a photograph. The reason of this is that the intensity and penetrative power of the rays, especially when the apparatus is being worked at its highest pitch, are constantly varying. At one moment the rays may be of just the quality which is necessary for the proper display of the object sought for, while at the next they may penetrate everything alike; and thus during the time while a photograph is being taken it may happen again and again that a good picture may be produced only to be wiped out by a succeeding set of rays to which everything is equally transparent. To the eye, however, these pictures, although evanescent, are visible at least for the moment, and thus it is possible with the screen for the observer to seize upon the moment when there is some slight difference in the degree of penetration, and when a shadow is thrown for an instant upon an otherwise bright field, and thus to take note of a condition which in a photograph would be entirely lost. A photograph does but represent the sum of a series of impressions, and if the exposure is at all prolonged it is quite possible for a sudden increase in the penetrative power of the rays to obliterate all that has been produced before. A negative result, then, in the case of a photographic plate is not worth anything as evidence of the non-existence of the thing sought for. As to the detection of renal calculi, if the patient is thin and anæmic, and if the calculus is composed of oxalate of lime, positive evidence can usually be obtained. Again, tuberculous deposits in the kidney are never sufficiently opaque to throw a definitely limited shadow like that of a calculus, so that the diagnosis between these conditions has been in some cases settled at once. In some cases it has been shown that in addition to the main calculus others have existed buried in the substance of the kidney, even so deeply that they might have been passed over at the time of operation. Phosphatic calculi are harder to detect, and those of uric acid or of urates harder still, the absorptive capacity of a uric acid calculus being scarcely greater than that of the highly vascular kidney which surrounds it, and when a patient is stoutly built, and especially when he is muscular as well as stout, success is still more rare. In all cases it is evident that what is important is positive rather than negative evidence.

